# Economic Inequality and Masculinity–Femininity: The Prevailing Perceived Traits in Higher Unequal Contexts Are Masculine

**DOI:** 10.3389/fpsyg.2019.01590

**Published:** 2019-07-30

**Authors:** Eva Moreno-Bella, Guillermo B. Willis, Miguel Moya

**Affiliations:** Department of Social Psychology, University of Granada, Granada, Spain

**Keywords:** economic inequality, masculinity, femininity, stereotypes, social class

## Abstract

Previous studies have shown that economic inequality influences psychological processes. In this article, we argue that economic inequality also makes masculine attributes more prototypical. In Study 1 (*N* = 106), using an experimental design, we showed that individuals belonging to a society characterized by a higher level of economic inequality are perceived as more masculine than feminine. Study 2 (*N* = 75) shows, also experimentally, that the upper social class is perceived mostly in terms of masculine traits, and that this effect is greater when economic inequality is relatively high. Conversely, the lower social class is more clearly perceived in terms of feminine traits. These results inform our understanding of the impact of economic inequality on social perception.

## Introduction

In recent years, economic inequality has been growing in the majority of developed countries (Piketty and Saez, 2014). This inequality is associated with important psychological processes, the most unequal societies tend to promote relational dynamics that are focused on personal independence and individualism (Sánchez-Rodríguez et al., 2018, 2019), competitiveness (Sánchez-Rodríguez et al., 2018; Sommet et al., 2018), and aggressiveness and hostility (Greitemeyer and Sagioglou, 2017).

In this article, we assert that these characteristics – independence, competitiveness, and aggresiveness – of more unequal societies may generate a stereotype for their individual members, i.e., they generate some expectations about individuals’ more representative characteristics or attributes. More precisely, we state that, in the most unequal societies, stereotypes are associated with attributes that have, traditionally, been defined as masculine.

### Psychosocial Effects of Economic Inequality

Economic inequality influences social psychological processes (Buttrick and Oishi, 2017; Wilkinson and Pickett, 2017). The rationale behind how economic inequality has psychosocial effects is the notion that different social structures provide different environments, which are fundamental to the development of human characteristics (Wilkinson and Pickett, 2017). It is therefore crucial to be aware of the extent to which a given society is hierarchical or egalitarian. In terms of distribution of resources and income, it may be necessary to determine which social strategy is appropriate in an unequal context, relative to a more egalitarian one: in a more unequal context, competition and dominance are social strategies that seem appropriate; in more equal contexts, strategies based on reciprocity and cooperation seem more suitable (Wilkinson and Pickett, 2017). Consistent with this idea, Buttrick and Oishi (2017) have argued that economic inequality influences society by increasing individuals’ mistrust and anxiety about their own positions.

From this perspective, experimental social–psychological studies have corroborated some of the proposed causal effects of economic inequality. For instance, economic inequality has been found to influence perceived societal norms, leading individuals to infer that others are more individualistic (Sánchez-Rodríguez et al., 2018); it also leads them to describe themselves by means of an independent self-construal (Sánchez-Rodríguez et al., 2019). At the same time, economic inequality is an important predictor of perceived competitiveness within a society (Sánchez-Rodríguez et al., 2018; Sommet et al., 2018). When people perceive that there is a high degree of economic inequality, they tend to be less cooperative with others (Nishi et al., 2015), and high status people become less generous [Côté et al., 2015; but see Schmukle et al. (2019) for a non-replication of this finding]. Similarly, when individuals face a disadvantageous situation in a context of economic inequality, they tend to be more aggressive toward others (Greitemeyer and Sagioglou, 2017).

In this paper, we are interested in the consequences of perceived economic inequality on societal inferences about others. Given that many of the above characteristics – independence, competitiveness, and aggressiveness – have commonly been associated with social constructions of masculinity (see Bem, 1974), we suggest that individuals living in societies that are perceived as more unequal may be perceived in terms of traits more closely associated with masculinity (and less associated with femininity).

### Masculinity–Femininity

Masculinity and femininity are cultural constructs related to gender (Bem, 1993; Starr and Zurbriggen, 2017), which can be applied to both subjects and groups (Ellemers, 2018). The masculinity construct, traditionally linked to men, is understood as the gender dimension involving the characteristics associated with carrying out work (Mehta and Dementieva, 2017), acting as a leader and being self-sufficient, independent, or aggressive (Parsons and Bales, 1955; Bem, 1974; Bem et al., 1976; Prentice and Carranza, 2002; Moya, 2003; Berdahl et al., 2018). By contrast, femininity, traditionally associated with women, includes attributes related to attending to others’ well-being (Mehta and Dementieva, 2017), understanding others, or being sensitive to others’ needs, inter alia (Parsons and Bales, 1955; Bem, 1974; Bem et al., 1976; Prentice and Carranza, 2002; Moya, 2003).

In the field of social psychology, masculinity and femininity have been equated with other gender constructs, with which they share a common core, given that they also represent an orientation toward the achievement of goals (*agency*, *instrumentality*, and *competence*) or toward the preservation of good relationships between members of a group (*communion*, *expressiveness*, and *sociability*) (Parsons and Bales, 1955; Spence and Helmreich, 1980; Abele and Wojciszke, 2014; Mehta and Dementieva, 2017). Masculine and feminine traits represent those behaviors that are expected from men and women, respectively; they function as prescriptive gender stereotypes (Prentice and Carranza, 2002). These expectations about what should be done to belong to a group of men or women reinforce and justify the gender roles and inequality between both sexes (Ellemers, 2018).

In much the same way as people, societies can be more or less feminine. For example, Hofstede’s research shows that societies can be categorized according to five dimensions; one such dimension is masculinity/femininity, by which it is possible to determine the extent to which societies are focused on achieving self-oriented objectives (masculine societies), relative to social objectives (feminine societies) (Hofstede, 1998). In those societies labeled as masculine, there is a bigger differentiation of gender roles, relative to those societies labeled as feminine. In fact, in feminine societies, both men and women care for the improvement of living standards and are not focused on financial success (Hofstede, 1991, 1998).

### The Current Research

In the present research, we examine how individuals within a given society are perceived by others, in terms of the traits that form the social constructs of masculinity and femininity, and according to the society’s level of economic inequality. Specifically, in Study 1, we predicted that when the level of economic inequality is relatively high, then average members of that society are perceived as more masculine than feminine (H1).

In Study 2, we examined this relation, but added another key variable: perceptions about upper and lower social class members. Previous studies examined whether there are differences between the ways that social classes are perceived. Fiske et al. (2002), in their research about the stereotype content model (SCM), suggest that social classes are perceived stereotypically, but with ambivalence about the dimensions of *competence* (related to masculinity) and *warmth* (related to femininity; Abele and Wojciszke, 2014, 2019). Upper-class individuals are perceived as competent but cold, whereas lower-class individuals tend to be perceived as warm but incompetent (Fiske et al., 2002; Durante et al., 2013, 2017). Thus, in Study 2, we examined whether: (a) members of the lower social class are regarded as more stereotypically feminine than masculine, notwithstanding the level of economic inequality (H2); and (b) members of the upper social class are regarded as stereotypically more masculine than feminine, notwithstanding the level of economic inequality (H3).

Finally, we also hypothesize that increasing inequality increases the perception of upper social class individuals of the society as more masculine than feminine (H4). We did not have a clear prediction, however, about the effects of inequality on perceptions of lower-class individuals. On the one hand, it can be argued that inequality also increases perceptions of masculinity about this group (for the same reasons we have argued that inequality has a main effect on the average member). On the other hand, economic inequality has also been found to increase the ambivalence of perceptions (Durante et al., 2013), so that groups tend to be perceived in a more polarized way within their stereotype. From this perspective, it is reasonable to expect that low status groups are perceived as more feminine than masculine when inequality is high. In Study 2, we explore which of these two impacts of economic inequality bear on the masculinity/femininity of lower-class individuals.

## Study 1

In this study, we investigated how the average member of a society with higher economic inequality (as opposed to lower inequality) is described, in terms of masculinity and femininity. We expected, according to H1, that the average member of a society with high economic inequality (vs. another society with lower inequality) would be described as more masculine than feminine.

### Materials and Methods

#### Participants and Procedures

Participants volunteering for the study included 106 psychology students from the University of Granada, of whom 89 were women and 17 were men, whose average age was 21.87 years (*SD* = 3.84). The participants were randomly assigned to each experimental condition.

We conducted a sensitivity power analysis. For a mix-design ANOVA (with two groups and two dependent variables), this sample size (*N* = 106) allows us to detect an effect size as small as *f* = 0.22 ($\eta_{p}^{2}$ = 0.05) with a power of 0.80 (and an alpha level set at 0.05).

This study’s sample was collected in the Psychology Faculty of the University of Granada. The students were offered university credits in the subject of Social Psychology. Also, the researcher received informed written consent from the participants, who read information about their voluntary participation, as well as the anonymity and confidentiality of their answers. The researcher explained the instructions to all participants and handed out paper copies of the questionnaires, ensuring that each had enough time to complete the questionnaire during class time. The study was conducted after receiving approval from the Ethics Committee of the University of Granada.

#### Materials

##### Manipulation of economic inequality

The participants read a text about an extraterrestrial society, in which the inhabitants are neither women nor men, following a similar approach to that taken by Hoffman and Hurst (1990). The text briefly described this society, and finally, stated that, as in other societies, this one was stratified on the basis of unequal distribution of resources to various groups; the salary of the 10% with the highest income was either 30 times higher than the earnings of the poorest 10% of the population (condition of higher economic inequality), or 5 times higher (condition of lower economic inequality) (see Supplementary Material S1). After reading the text, they were given a manipulation check, which asked participants: “Please, answer to which extent you think that income distribution in the extraterrestrial society is unequal.” The answer choices consisted of a scale, which ranged from 0 (*not at all*) to 100 (*very much*), in 10-point increments.

##### Masculinity–femininity of the average member

The participants were asked to consider the nature of the average member of the extraterrestrial society. To assess their answers, we used the items of the Spanish adaptation of the Bem Sex Role Inventory (BSRI) (Bem, 1974; adapted to Spanish by Páez and Fernández, 2004). This inventory consists of 18 items, of which 9 measure the social construct of masculinity (α = 0.83) – for example, “strong personality,” “acting as a leader,” and “dominant” – and the other 9 items measure the social construct of femininity (α = 0.87) – “sensitive to others’ needs,” “loving,” and “loves children” (see Supplementary Material S2). In this case, the answer format was a seven-point Likert scale ranging from 1 (*not at all*) to 7 (*very much*); however, unlike the BSRI, where the participants must think of themselves and provide a personal answer, in this case, participants had to indicate whether they considered every trait characteristic for the average extraterrestrial member of the hypothetical society.

##### Political orientation

The participants indicated their political orientation by answering to the following item: *In politics, it is often discussed that there is “left-wing” and “right-wing.” Where would you place yourself on a scale in which 1 means “left-wing” and 10 means “right-wing?*”

##### Social class

We then assessed subjective social class, according to a MacArthur Scale of Subjective Social Status (Adler et al., 2000). Participants were also asked about family income level. The family income level was assessed using scale, consisting of 10 intervals, within which they had to appropriately situate their monthly family income.

##### Sociodemographic data

We asked participants about their gender, age, nationality, and educational attainment. They had to state their educational attainment according to an eight-category scale, on which the lowest level was “elementary studies” and the highest was “Ph.D. studies.”

### Results

The results obtained from the manipulation check showed that there were differences in the experimental conditions: the participants assigned to a condition of higher economic inequality (*M* = 76.59; *SD* = 16.59) perceived a higher level of inequality for income distribution in that society than in the other experimental condition (*M* = 51.84; *SD* = 23.42), *F*(1,94) = 35.45, *p* < 0.001, $\eta_{p}^{2}$ = 0.27.^1^

**FIGURE 1:**
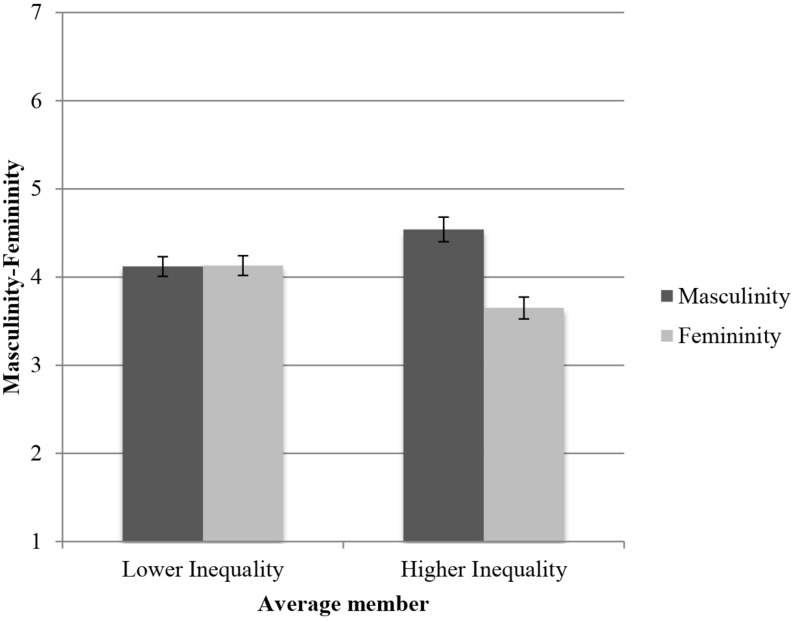
Average rating in masculinity–femininity of the average member according to the level of economic inequality of the society.

Next, we tested our main hypothesis, using a mixed-design ANOVA of 2 (Economic inequality: Higher inequality vs. Lower inequality) × 2 (Masculinity vs. Femininity), with repeated measures in the last variable. We tested simple effects within the same ANOVA, adjusting for multiple comparisons (Bonferroni). Results showed a main effect of the masculinity–femininity variable, *F*(1,104) = 9.62, *p* = 0.002, $\eta_{p}^{2}$ = 0.08. The participants generally evaluated the average extraterrestrial member as more prototypically masculine [*M* = 4.33, 95% CI = (4.15, 4.51)] than feminine [*M* = 3.89, 95% CI = (3.73, 4.06)], *M*_*D*_ = 0.44, 95% CI = (0.16, 0.72). However, this effect was qualified by a significant interaction between masculinity–femininity and economic inequality, *F*(1,104) = 9.95, *p* = 0.002, $\eta_{p}^{2}$ = 0.09 (Figure 1). In the condition of lower inequality, the average extraterrestrial member was evaluated using, both masculine [*M* = 4.13, 95% CI = (3.87, 4.38)] and feminine [*M* = 4.13, 95% CI = (3.90, 4.37)] traits, *M*_*D*_ = −0.01, 95% CI = (−0.40, 0.39); *F* < 1. By contrast, and corroborating H1, in the condition of higher inequality, the average extraterrestrial member was assessed as more masculine [*M* = 4.54, 95% CI = (4.28, 4.79)] than feminine [*M* = 3.65, 95% CI = (3.42, 3.89)], *M*_*D*_ = 0.88, 95% CI = (0.49, 1.28); *F*(1,104) = 19.57, *p* < 0.001, $\eta_{p}^{2}$ = 0.16.

Finally, we conducted a robustness check by running a mix-design ANCOVA, using the same dependent and independent variables, but using as covariates: political orientation, subjective social class, sex, age, income level, and educational attainment. The main results were not influenced by these sociodemographics variables (see Supplementary Material S3). Means, standard deviations, and correlations are available in Supplementary Material S4.

### Discussion

In this study, we found that, when the economic inequality of a society is higher, the average member of that society is perceived with more masculine than feminine traits, which verifies our H1. Nonetheless, we found no differences in the evaluation of an average member of a society characterized by conditions of lower economic inequality. This suggests that, in a society where economic inequality is lower, individuals may be perceived as less different from each other, or, as sharing more similar traits. It may be inferred that, in a situation of lower economic inequality, individuals may be perceived in more androgynous terms.

We performed another study to further research how members of a society are perceived according to the level of economic inequality. Here, we assessed the perception of members of the upper and lower social classes within a society, according to the existing level of economic inequality.

## Study 2

Given that social class triggers certain stereotypes (Fiske et al., 2002; Fiske, 2005; Piff et al., 2018), we were interested to know the moderating role of the targets’ social class in the relationship between economic inequality and perceived masculinity–femininity. Therefore, in this study, we tried to check how the members of the upper and lower social classes of an imaginary society are perceived, according to the level of economic inequality (Higher inequality vs. Lower inequality) (see H2, H3, and H4).

### Materials and Methods

#### Participants and Procedures

In this second study, however, we were only able to recruit 75 Psychology students from the University of Granada (59 women and 16 men), with an average age of 24.48 years (*SD* = 2.03). The participants were randomly assigned to one of the two experimental conditions.

To determine the effect size we conducted a sensitivity power analysis. The present sample size (*N* = 75) allows us to detect an effect size as small as *f* = 0.22 ($\eta_{p}^{2}$ = 0.05), considering a statistical power of 0.80 (and an alpha level set at 0.05) for a mix-design ANOVA (with two groups and four dependent variables).

This study’s sample was collected in the Psychology Faculty of the University of Granada. The students were offered university credits in the subject of Social Psychology. As in Study 1, the researcher received informed written consent from the participants, who read information about the voluntary nature of their participation, as well as the guaranteed anonymity and confidentiality of their answers. The researcher explained the instructions to all the participants and handed out paper copies of the questionnaires, ensuring they each had enough time to fill them in the questionnaires during class time. The study was conducted after receiving approval from the Ethics Committee of the University of Granada.

#### Materials

##### Manipulation of economic inequality

The manipulation of economic inequality was operated in the same way as in Study 1. Participants assigned to the higher economic inequality condition read the text about the fictitious society, in which the salary of the 10% with the highest income was either 30 times higher than the earnings of the poorest 10% of the population; and participants assigned to the lower economic inequality condition read that the differences of salary between the 10% with the highest income was 5 times greater than the poorest 10% of society. After the economic inequality manipulation, participants completed the manipulation check in which they indicated the degree they considered that society was unequal, on a range from 0 (*not at all*) to 100 (*very much*), as in Study 1.

##### Masculinity–femininity of typical upper and lower-class members

We used the Spanish version of BSRI (Bem, 1974; adapted to Spanish by Páez and Fernández, 2004), as in Study 1. However, in this study, the participants assessed two extraterrestrial members using this scale: one belonged to the upper social class (with more resources) and the other to the lower social class (with fewer resources). The reliability of masculinity when they were assessing the upper-class extraterrestrial member was α = 0.75; when they assessed the lower-class member, reliability was α = 0.75. The reliability of femininity was α = 0.89 and α = 0.83 for the extraterrestrial member’s assessment, according to upper and lower social class, respectively.

##### Political orientation

The measure used in Study 1 to assess the political orientation was applied here.

##### Social class

To assess the social class, we used the same measure (Adler et al., 2000) as in Study 1. We also asked participants about their family income level using the scale of 10 intervals as in Study 1.

##### Sociodemographic data

The participants stated their age, nationality, and educational attainment in the same way they did in Study 1, using the same answer format for the answers.

### Results

By analyzing the data obtained from the manipulation check, we were able to observe that, as in Study 1, there were differences between the experimental conditions: the participants assigned to the condition of higher economic inequality perceived that the economic inequality was greater (*M* = 73.45, *SD* = 16.32) than the participants assigned to the condition of lower economic inequality (*M* = 48.95, *SD* = 22.39), *F*(1,65) = 24.67, *p* < 0.001, $\eta_{p}^{2}$ = 0.27.

A mixed-design ANOVA was subsequently performed, to test the different hypotheses (H2, H3, and H4), using a 2 (Economic inequality: Higher inequality vs. Lower inequality) × 2 (Social class: Upper social class vs. Lower social class) × 2 (Masculinity vs. Femininity) design, with repeated measures in the last two variables (Table 1). We tested simple effects within the same mixed-design ANOVA, adjusting for multiple comparisons (Bonferroni).

**TABLE 1:** Results from mix-design ANOVA: 2 (economic inequality: higher inequality vs. lower inequality) × 2 (social class: upper social class vs. lower social class) × 2 (masculinity vs. femininity).

	***F***	***p*-value**	$\eta_{p}^{2}$
MF	2.81	0.098	0.037
SC	1.47	0.229	0.020
MF × SC	167.83	0.000	0.697
EI × MF	5.11	0.027	0.065
EI × SC	2.66	0.107	0.035
EI × SC × MF	5.21	0.025	0.067

*MF, masculinity–femininity; SC, targets’ social class; EI, economic inequality.*

Following our prediction, we found a significant three-way interaction between economic inequality, the target’s social class, and masculinity–femininity, *F*(1,73) = 5.21, *p* = 0.025, $\eta_{p}^{2}$ = 0.07.

When this interaction was analyzed, we first found that, in both the high [*M*_D_ = 1.26, 95% CI = (0.96, 1.55); *F*(1,73) = 71.35, *p* < 0.001, $\eta_{p}^{2}$ = 0.49] and the low inequality condition [*M*_D_ = 1.16, 95% CI = (0.90, 1.42); *F*(1,73) = 77.61, *p* < 0.001, $\eta_{p}^{2}$ = 0.51], lower-class individuals were evaluated as more feminine than masculine (Table 2). This corroborates H2.

**TABLE 2:** Means, standard deviations, and 95% confidence intervals of results of mix-design ANOVA of Study 2.

**Higher economic inequality**							

**Upper social class member**				**Lower social class member**			
**Masculinity**		**Femininity**		**Masculinity**		**Femininity**	
***M* (*SD*)**	**95% CI**	***M* (*SD*)**	**95% CI**	***M* (*SD*)**	**95% CI**	***M* (*SD*)**	**95% CI**
5.12 (0.71)	[4.838, 5.398]	3.20 (0.82)	[2.882, 3.515]	3.70 (0.65)	[3.441, 4.959]	4.96 (0.65)	[4.704, 5.209]

**Lower economic inequality**							

**Upper social class member**				**Lower social class member**			
**Masculinity**		**Femininity**		**Masculinity**		**Femininity**	
***M* (*SD*)**	**95% CI**	***M* (*SD*)**	**95% CI**	***M* (*SD*)**	**95% CI**	***M* (*SD*)**	**95% CI**

4.93 (0.87)	[4.677, 5.173]	3.86 (0.98)	[3.582, 4.143]	3.79 (0.81)	[3.559, 4.018]	4.95 (0.78)	[4.726, 5.173]

*2 (Economic Inequality: Higher inequality vs. Lower inequality) x 2 (Social Class: Upper social class vs. Lower social class) x 2 (Masculinity vs. Femininity. Ratings were given on a 7-point scale from 1 (not at all) to 7 (very much).*

**FIGURE 2:**
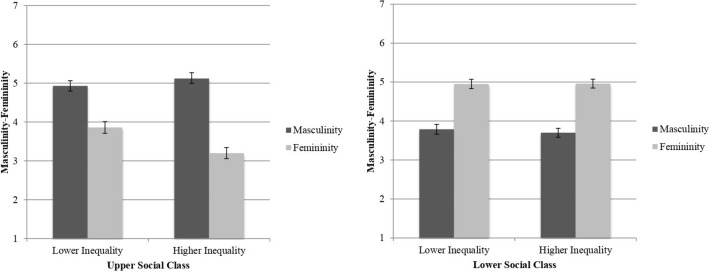
Average rating in masculinity–femininity according to the level of economic inequality and target’s social class.

In addition, and consistently with H3, we found that, in both the high [*M*_*D*_ = 1.92, 95% CI = (1.44, 2.40); *F*(1,73) = 63.16, *p* < 0.001, $\eta_{p}^{2}$ = 0.46] and in the low inequality conditions [*M*_*D*_ = 1.06, 95% CI = (0.64, 1.49); *F*(1,73) = 24.65, *p* < 0.001, $\eta_{p}^{2}$ = 0.25], upper social class members were perceived as more masculine than feminine (Table 2). Importantly, and as the significant three-way interaction suggests, this difference was greater in the higher economic inequality condition than in the lower economic inequality condition (Figure 2). Thus, as predicted in H4, we found that upper class individuals were evaluated using more masculine than feminine traits, especially when economic inequality was high (vs. lower inequality).

Apart from the main analysis, to verify the results, we conducted the same mixed-design ANCOVA, controlling for political orientation, subjective social class, sex, age, income level, and level of studies. The three-way interaction was not influenced by these variables (see Supplementary Material S5). Means, standard deviations, and correlations are available in Supplementary Material S6.

### Discussion

In this study, we found that, when participants evaluate upper social class individuals they consider masculine traits to prevail over feminine ones. Although lower social class individuals tended to be described by participants mostly through feminine traits, we did not found that this effect was influenced by economic inequality. In short, these results indicate that economic inequality only changes how individuals judge the prototypically advantaged members of society.

## General Discussion

These two studies provide us with information about the potential consequences of economic inequality on the perception of the members of a given society, in terms of their masculinity and femininity. In Study 1, we found that the perception of an average inhabitant of a society of which we know very little may vary, depending on the level of economic inequality characterizing that society. If the level of inequality is low, then the inhabitants are perceived as more similar, in terms of their masculinity and femininity. Nonetheless, if there is higher economic inequality, then the inhabitants of that society are perceived as more masculine than feminine. These results are consistent with previous research, which has found that the most stereotypical traits of masculinity, such as independent self-construal (Sánchez-Rodríguez et al., 2019), competitiveness (Sánchez-Rodríguez et al., 2018; Sommet et al., 2018), and aggressiveness (Greitemeyer and Sagioglou, 2017) are associated with economic inequality.

It is important to note that our research has shown that there are differences in the way we perceive others, in terms of traditional gender traits, according to economic inequality, without having offered information about the gender of the members of the society. Besides, as in Study 1, the typical member of the society was evaluated with more masculine than feminine traits in the society with higher economic inequality; this may be interpreted as reflecting that, in a higher-inequality context, people regarded central or core traits as masculine, relative to feminine traits. Conversely, under not-so-high – or lower – inequality conditions, the core traits seem to be equally masculine and feminine. In addition, in Study 2, upper-class members were perceived as representing the same core traits that we found in Study 1 – that is, masculine traits – especially in the context of greater economic inequality. These results may be understood on the basis of the notion that the valued or core traits are associated with dominant groups (Tajfel, 1981; Jost and Banaji, 1994; Pratto et al., 1994; Sidanius and Pratto, 1999; Correll and Ridgeway, 2003; Ho et al., 2012; Cuddy et al., 2015), and because men are the dominant group regarding gender (Glick et al., 2000; Fiske et al., 2002, 2016), it is reasonable to expect masculine traits to be reflected as the culture’s most valued traits (relative to feminine traits), especially under more unequal conditions.

In Study 2, we extended the research by including an assessment of members of upper and lower social classes. Upper-class individuals, notwithstanding the level of economic inequality, were perceived as prototypically more masculine than feminine (H3), whereas lower-class subjects, notwithstanding the level of economic inequality, were perceived as more feminine than masculine (H2). Our results extend the literature about the way different social classes are perceived (see Fiske et al., 2002). Moreover, members of upper social classes are perceived as more prototypically masculine when the level of inequality is higher, relative to when it is lower (H4). We did not find, however, that the level of inequality influenced how lower-class members are evaluated. Economic inequality may have influenced the perception of this group in two contrasting ways, and these may have canceled each other out. On the one hand, it may be that, for some participants, inequality increases perceived masculinity for all the members of the society; for others, however, inequality may increase the ambivalence of their perceptions, prompting them to evaluate lower-class members as even more feminine than masculine.

However, it is important to be cautious when interpreting Study 2’s three-way interaction because of (a) the relatively small sample size of Study 2 and (b) the fact that interactive effects are typically harder to replicate than main effects (Altmejd et al., 2019).

Our findings have implications for the research on gender, social class, and economic inequality. The contribution of our research to the wide literature on stereotypes and social classes is the discovery that a society’s level of economic inequality is a relevant variable that specifically affects masculine traits. Given that our results suggest that, in more economically unequal societies, traditionally masculine traits are more representative than feminine traits, we reckon that this may negatively affect women, who are generally expected to behave according to those traits that are traditionally more feminine than masculine. In this respect, existing literature has already tested that, in prototypically masculine contexts – or rather, in the contexts where masculine traits are the most representative ones, as is the case of leadership – women are disadvantaged solely because they are women, provided that they are expected to adapt their behavior to their predefined roles (Eagly and Karau, 2002; Ryan et al., 2011). Further, the richest people in the world are mostly men – according to the perception of the upper social class as prototypically masculine (H2), whereas the majority of world’s poor are women – the *feminization of poverty* (Chant, 2007; Gornick and Boeri, 2016). That is to say that there is a connection between lower social class and femininity (H2), as we proposed. Additionally, economic inequality and gender inequality are not only closely related with each other (Seguino, 2010; Aslan et al., 2017; Deléchat et al., 2018), but it is also the case that, in countries with higher gender inequality, there are more men who support hostile sexist ideologies, thereby driving women to support benevolent sexist ideologies (Glick et al., 2000). Due to these relationships, it can be interpreted – and addressed in future research – that high levels of economic inequality may facilitate the maintenance of sexist ideologies and traditional gender stereotypes, at the expense of men and women, in comparison with low levels of economic inequality.

This research manifests some limitations. One was the sample type of both studies, composed of psychology students. The results could benefit substantially from our using general population samples, given that university students, especially those related to the field of psychology, may be more aware of gender stereotypes than other sectors of general society. Notwithstanding that limitation, even within this population with a high level of awareness, the results clearly indicate the existence of stereotypical perceptions, according to the level of inequality, which suggests that the effects within sectors of the population with lower awareness of gender inequality may be even more noticeable (e.g., Moya et al., 2000). We believe that another limitation of our research is that the samples of both studies were composed mainly of women, which may have influenced the results. In addition, although the two present experiments had high internal validity, we are aware that they suffer from a lack of ecological validity, which demands that we should be particularly cautious in drawing conclusions concerning real-world applications. Moreover, some reported effect sizes in our investigation are very large, especially in Study 2. This is consistent with related literature (e.g., Durante et al., 2017; Hentschel et al., 2019) and with the notion that mixed-designs tend to have smaller error terms, relative to between-groups design, which is associated with larger effect size (Raskin and Kircher, 2014).

In sum, with this research, we have tried to address some of the psychosocial consequences of economic inequality. The results presented here support and contribute to the evidence that economic inequality has an impact, at a psychosocial level, on the very way we perceive the others, according to the social constructs of gender (Masculinity vs. Femininity). Specifically, our results suggest that the level of economic inequality may be a strong macro-social factor influencing the prevalence of masculine traits, over feminine ones, as core traits in an economically unequal society.

## Data Availability

The dataset, syntax, and manipulation of our experiments are available at osf.io/xwd8r.

## Ethics Statement

This research, which was carried out in accordance with the guidelines stated by the Vicerectory of Research and Scientific Policy of the University of Granada, is part of a research project approved by the Ethics Committee of the University of Granada.

## Author Contributions

EM-B, GW, and MM designed the studies, analyzed and interpreted the data, and wrote the manuscript. EM-B conducted the studies.

## Conflict of Interest Statement

The authors declare that the research was conducted in the absence of any commercial or financial relationships that could be construed as a potential conflict of interest.

## Footnotes

1 Degrees of freedom vary given that we had missing values in some questions.
