# Supplementary material for: Economic Inequality and Masculinity–Femininity: The Prevailing Perceived Traits in Higher Unequal Contexts Are Masculine
Source: Front Psychol. 2019 Jul 30;10:1590. doi: 10.3389/fpsyg.2019.01590 (PMC6688552; doi:10.3389/fpsyg.2019.01590)
Supplement: Supplementary file 1 [file Data_Sheet_1.pdf]

Supplementary Material

Economic Inequality and Masculinity-Femininity: The Prevailing Perceived Traits in Higher Unequal Contexts Are Masculine

Eva Moreno-Bella, Guillermo B. Willis* and Miguel Moya

***Correspondence:** gbwillis@ugr.es

S1

Manipulation of Economic Inequality (used in Study 1 and Study 2). We present here a translation from the original stimulus (that were in Spanish).

"Imagine there is an extraterrestrial society in which the inhabitants cannot be considered either women or men. Their reproductive system is neutral, so anybody can breed with any other individual. This society has existed for centuries. The individuals are scaly-skinned and have two rows of teeth, and the rest of the attributes vary considerably among the inhabitants.

The members of this society enjoy their leisure time and have several opportunities to enjoy themselves, both with relatives and friends. Thanks to the development of new technologies, they have new ways to spend their spare time. The technological development is so advanced that they have managed to build a spaceship that allows them to travel at the speed of light. They also respect their cultures and traditions: they usually hold several festivals and yearly celebrations, but the most important one is that of New Year's Eve.

This society, as most are, is stratified: some groups of inhabitants can count on more resources than others. For instance, in this society, the richest 10% earns 30 times (5 times) as much as the poorest 10%. As a matter of fact, the differences within social groups are (are not) extremely high, for opportunities to access higher education, medical care or decent housing.

After reading the information about this extraterrestrial society, we want you to imagine what these inhabitants are like. How do they look? What are their characteristics? How are their personalities?

You will now be asked a series of questions about these individuals."

S2

Spanish adaptation of the BSRI (Bem, 1974; adapted to Spanish by Páez and Fernández, 2004), used in Study 1 and Study 2:

(Masc = Masculine trait; Fem = Feminine trait)

1. Atlético/a, deportivo/a (Masc)
2. Cariñoso/a (Fem)
3. Personalidad fuerte (Masc)
4. Sensible a las necesidades de los demás (Fem)
5. Desea arriesgarse, amante del peligro (Masc)
6. Comprensivo/a (Fem)
7. Compasivo/a (Fem)
8. Dominante (Masc)
9. Cálido/a, afectuoso/a (Fem)
10. Tierno/a, delicado/a, suave
11. Agresivo/a, combativo/a (Masc)
12. Actúa como líder (Fem)
13. Individualista (Masc)
14. Amante de los niños (Fem)
15. Alguien que llora fácilmente (Fem)
16. Duro/a (Masc)
17. Sumiso/a (Fem)
18. Egoísta (Masc)

S3

Main analysis of Study 1, controlling for sociodemographic variables.

To verify the results of Study 1, we conducted a mixed-design ANCOVA of 2 (Economic inequality: Higher vs. Lower) x 2 (Masculinity vs. Femininity). Economic inequality was the between-groups variable and Masculinity-Femininity was the within-participants variable, and political orientation, subjective social class, sex, age, income level, and educational attainment were covariates. We adjusted for multiple comparisons (Bonferroni). We did not find an effect of any covariates. Unlike the analysis without control variables, we did not found a main effect of Masculinity-Femininity, $F < 1$. There were no differences between masculinity ($M = 4.31$, $SD = .94$) and femininity ($M = 3.91$, $SD = .87$) of the average member.

Even so, we did find the interaction effect between masculinity-femininity and economic inequality, $F(1, 92) = 8.27$, $p = .005$, $\eta^2_p = .082$. In the lower economic inequality condition, there was no difference between the way the average member was perceived, masculine or feminine ($M_{masc} = 4.13$, 95% CI =[3.89, 4.40] vs. $M_{fem} = 4.16$, 95% CI = [3.92, 4.40]), $M_D = -.017$, 95% CI = [-.42, 38]; $F < 1$; but we found, in the higher economic inequality condition, that the average member was assessed as more masculine ($M = 4.48$, 95% CI = [4.21, 4.75]) than feminine ($M = 3.66$, 95% CI = [3.41, 3.90]), $M_D = .824$, 95% CI = [.41, 1.23]; $F(1, 92) = 15.99$, $p < .001$, $\eta^2_p = .15$. Thus, including covariates in these analyses did not change the main conclusion of this study.

S4

Table S1. *Means, standard desviations, and correlations of Study 1.*

Variables	*M* (*SD*)	1	2	3	4	5	6	7
1. Masculinity	4.33 (.94)	-						
2. Femininity	3.89 (.88)	-.359***	-					
3. Political Orientation	3.93 (1.75)	.005	-.047	-				
4. Subjective Social Class	5.99 (1.29)	-.038	-.098	.112	-			
5. Sex	.84 (.37)	-.071	.051	.087	.179	-		
6. Age	21.87 (3.84)	.002	.010	-.020	-.039	-.170	-	
7. Income Level	4.51 (2.03)	.086	-.053	.145	.672***	.073	-.061	-
8. Educational Attainment	5.04 (.31)	.231*	-.095	.094	-.102	-.199*	.118	-.031

Note. *$p < .05$, **$p < .01$, ***$p < .001$

S5

Main analysis of Study 2, controlling for sociodemographics variables.

We decided to perform a mixed-design ANCOVA of 2 (Economic inequality: Higher vs. Lower) x 2 (Social class: Upper Social Class vs. Lower Social Class) x 2 (Masculinity vs. Femininity). Economic inequality was the between-groups variable, whereas Social Class and Masculinity-Femininity were the within-participants variables, and political orientation, subjective social class, sex, age, income level, and educational attainment were covariates. We adjusted for multiple comparisons (Bonferroni). We found an effect of the following covariates: subjective social class (F(1, 67) = 5.80, p = .019, η^2_p = .08), income level (F(1, 67) = 4.161, p = .045, η^2_p = .058), and educational attainment (1, 67) = 10.26, p = .002, η^2_p = .13.This analysis yielded a significant three way interaction between economic inequality, social class and masculinity-femininity, F(1, 67) = 5.41, p = .027, η^2_p = .07. We analyzed this interaction and we corroborated that, in higher (M_D = 1.281, 95% CI = [.99, 1.57]; F(1, 67) = 77.30, p < .001, η^2_p = .54) and in lower (M_D = 1.14, 95% CI = [.88, 1.40]; F(1, 67) = 78.42, p < .001, η^2_p = .54) economic inequality conditions, lower social class individuals were assessed as more feminine than masculine (see Table 5); as in the analysis without control variables and corroborating H2. Moreover, we confirmed that in the higher (M_D = 1.89, 95% CI = [.1.42, 2.36]; F(1, 67) = 64.68, p < .001, η^2_p = .49) and the lower economic inequality conditions (M_D = 1.08, 95% CI = [.67, 1.50]; F(1, 67) = 27.08, p < .001, η^2_p = .29), upper social class individuals were perceived as more masculine than feminine, as we predicted in H3. This difference was greater in the higher economic inequality condition than in the lower economic inequality condition (H4). Thus, and as in Study 1, including these covariate did not change the main conclusions of the study. See the table below for means, standard deviations and 95% confidence intervals (Table S2).

Table S2.

Means, standard deviations, and 95% confidence intervals of results of mix-design ANCOVA of Study 2.

Higher Economic Inequality							
**Upper Social Class Member**				**Lower Social Class Member**			
**Masc**		**Fem**		**Masc**		**Fem**	
***M* (*SD*)**	**95% CI**	***M* (*SD*)**	**95% CI**	***M* (*SD*)**	**95% CI**	***M* (*SD*)**	**95% CI**
5.12 (.71)	[4.84, 5.40]	3.23 (.82)	[2.92, 3.53]	3.70 (.65)	[3.43, 4.73]	4.98 (.65)	[4.73, 5.24]

Lower Economic Inequality							
**Upper Social Class Member**				**Lower Social Class Member**			
**Masc**		**Fem**		**Masc**		**Fem**	
***M* (*SD*)**	**95% CI**	***M* (*SD*)**	**95% CI**	***M* (*SD*)**	**95% CI**	***M* (*SD*)**	**95% CI**
4.92 (.87)	[4.67, 5.17]	3.84 (.98)	[3.57, 4.11]	3.79 (.81)	[3.55, 4.03]	4.93 (.78)	[4.70, 5.15]

Note. 2 (Economic Inequality: Higher inequality vs. Lower inequality) x 2 (Social Class: Upper social class vs. Lower social class) x 2 (Masculinity vs. Femininity). Masc = Masculinity; Fem = Femininity. Ratings were given on a 7-point scale from 1 (*not at all*) to 7 (*very much*).

S6

Table S3. *Means, Standard Desviations, and Correlations of Study 2.*

Variables	*M* (*SD*)	1	2	3	4	5	6	7
1. Masculinity	4.38 (.56)	-						
2. Femininity	4.26 (.67)	.283*	-					
3. Political Orientation	3.33 (1.78)	.127	.055	-				
4. Subjective Social Class	5.86 (1.58)	.027	.276*	.232*	-			
5. Sex	.79 (.41)	-.012	-.006	-.049	-.065	-		
6. Age	21.48 (2.03)	.048	.104	.165	.079	-.102	-	
7. Income Level	4.69 (2.27)	.061	.053	.092	.602***	-114	-.062	-
8. Educational Attainment	4.97 (.57)	.194	-.176	.018	-.144	.008	.128	-.254

Note. $^*p < .05$, $^{**}p < .01$, $^{***}p < .001$.
